# Ununited Fractures

**Published:** 1895-07

**Authors:** J. R. Stuart

**Affiliations:** Houston, Texas


For the Texas Medical Journal.

### UNUNITED FRACTURES.

BY J. R. STUART, M. D., HOUSTON, TEXAS.

[Read at meeting of Houston District Medical Society, 1895.]

UNUNITED fractures are of interest to the profession from a professional standpoint, from a legal standpoint, and from a pecuniary point of view. Professionally, our pride is wounded if one of our cases results in this manner; legally, we are liable to be sued for malpractice for having such a result; pecuniarily, patients object to paying physicians unless their work gives satisfaction.

For these three reasons, therefore, we should endavor to prevent their occurrence. In the event, however, that they do occur in our practice, then we should prepare to treat them scientifically. Fortunate, indeed, is the fact that these cases seldom occur; I believe the proper ratio is about one case of non-union to every one thousand fractures observed.

It is not my purpose to discuss in general the subject of ununited fractures, but merely to mention the cause and treatment, concluding by exhibiting to you a patient recently under treatment at the Houston Infirmary for non-union of a fractured femur.

The subject of non-union in fractures is an old one, it having afforded entertainment for the surgeon ever since the first article was written on the subject in 1760. All surgeons, all general practitioners, who have been long in the harness, at some time during their professional career are surprised, astonished, and, I may add, chagrined, when, after removing the dressing from a fractured limb of a patient, watched tenderly and carefully for six long weeks, and from whom they confidently expected a large fee and a good result, the discovery is made that one more joint exists than is allowed by mother nature. If the patient retains him in charge, the perplexed physician turns to his surgery, to surgical literature, and to any other source whence he may derive information as to the further management of his patient, and solace to his wounded vanity.

The investigation develops that a non-united fracture may originate from the following causes: 1. Syphilis. 2. Mobility of the fragments. 3. Meddlesome interference on the part of the physician himself. 4. Injury to the periosteum. 5. Presence of pus in compound fractures. 6. Interposition of a substance between the fragments, such as blood clot or muscle. 7. Imperfect apposition. 8. Injury to the nutrient vessels. 9. Injury to the nerve trunks may delay union and occasionally prevent it. 10. Traumatisms of the spine involving the cord.

These, in general, constitute the causes of non-union in fractures. In all disabling injuries, the sufferer should be supplied with tonics, stimulants and a nutritious diet; he should be made as comfortable as the circumstances will allow; his bed should be kept clean and changed| not less than once a week; his room should be thoroughly ventilated, and all articles that contain germs, dust or dirt, banished forthwith. If non-union is thought to be due to a defective dressing, the defect should be at once remedied. In all cases the cause should be found, if possible. and remedied by proper appliances. Blisters over the seat of fracture are said to be worthy of trial. Friction of the ends of the fragments against each other, causing an inflammatory action and exudation of callous, has given good results. In a case of non-union in a fractured leg, treated by Dr. D. F. Stuart in 1887, when other means failed, this method was tried with success, the parts uniting within eight weeks. If ligamentous union exists, then with a tenotome a subcutaneous division of the ligaments has been advocated, and at the same time the edges of the fragments should be pared. Injections of irritating fluids, such as acetic acid, has also its advocates. I merely mention these, gentlemen, because they are in vogue; they are simple, and need not be discussed further than to say, that the results obtained are more or less unsatisfactory. Therefore, laying them aside, we come to the real purpose of this paper, and that is, resections as a means of relief in ununited fractures. As before stated, the first recorded instance of this operation is that it was performed by Dr. White, a surgeon residing in England in 1760. Very little more was said or thought of this surgical procedure until our civil war, when it was performed a number of times by surgeons of either side; and, considering the fact that the germ theory—the cause of pus, and its prevention and antisepsis had not been introduced or thought of—the results obtained were satisfactory. After, and until the discoveries of Lister and other scientists were noised abroad, it was allowed to again become dormant. Professor Gross refers to resections as very dangerous in their effect upon the patient. Antisepsis and modern surgical advancement has robbed of its terrors this once formidable operation, until now we regard it as a speedy and certain means of relief.

In the year 1892 I assisted Dr. Red at the county hospital in this city in performing the operation of resection. The patient, a negro named Tom Banks, aged thirty-five, was run over on the H. & T. C. road, resulting in a compound fracture of the right leg at the middle third. The injured soft parts healed nicely, but, to our surprise, upon removing the dressings, no union had taken place. After trying some of the methods enumerated above without success, recourse was had to the scalpel, the operation being performed in the usual manner. The patient recovered within eight weeks, and left the hospital without the aid of a cane or crutch. During the same year an engineer of the H., E. & W. T. R. R. was treated for a simple fracture of the leg, resulting in non-union; all of the methods referred to above were tried without success; he firmly and flatly refused to have the operation of resection performed, and finally changed physicians; the record of his case was therefore lost, and I do not know the result other than from what I have heard.

The patient we have here to-night, Chas. Williams, was employed, prior to the accident for which he has been treated, by the H., E. & W. T. R. R., in the train service department. On the 11th of October, 1894, while endeavoring to make a coupling, he was thrown between the cars, and the wheel of a flat car containing 60,000 pounds passed over his right thigh at the lower third. (You know, gentlemen, there are eight wheels to a flat car, and the entire weight in this case amounted to 68,000

pounds, and one-eighth of this, or about 8,000 pounds, must have rested on this poor fellow's thigh at one time.) The accident occurred at Lufkin, and resulted in a compound comminuted fracture. He was first seen by Dr. A. N. Denman, of the above town, who dressed the injured member, and afterwards removed four (4) inches of the bone from the shaft of the femur; Dr. Denman is an able surgeon, and a man of integrity, therefore I have no reason to doubt his word. Owing to the serious nature of the injury, it was deemed inexpedient to remove the patient; in consequence, he was allowed to remain under the care of Dr. Denman. The injury to the tissues healed rapidly, and very soon Dr. Denman was enabled to apply a fixed and permanent dressing. On the 21st of December, he was doing nicely, so nicely that he was regarded as being in a fit condition to move, and was accordingly ordered to the Houston Infirmary for further treatment.

He stood the fatigue incident to the journey remarkably well, and reached Houston in safety. Upon his arrival here, the fixed dressing applied by Dr. Denman was removed, and no union was discovered. As the fragments did not seem to be in apposition, it was decided to operate forthwith. The patient seemed to be in good physical condition; his family history showed no record of specific or pulmonary disease, and it was anticipated that by a carefully performed and judiciously managed operation, we would yet be able to make him a good leg. The preparations were similar to those in vogue in all modern operations: instruments, dressings, sutures. aprons and operating table were all sterilized; the field of operation was carefully shaved, and bathed first with a solution of corrosive sublimate, 1 to 2000, and then with sulphuric ether; hands of the surgeons and assistants were disinfected by rinsing in a solution of potass. permanganate and then in oxalic acid solution, finishing with sterilized water and absolute alcohol. The condition of the nails was also looked to, and all finger rings were banished from the operating hall. The patient was then chloroformed.

The primary incision was begun on the median line, on the anterior surface of the thigh, about six inches above the patella, and extended downward to terminate opposite the condyles of the femur. The incision was carried directly down to the fracture, when the following condition was observed: No callous at all had been exuded; the jagged ends of the femur appeared as they must have done when the injury was received. The upper fragment overlapped the lower by about half an inch, both fragments being firmly bound down by ligamentous union.

After some delay, and with the aid of an instrument devised by Prof. Wyeth (that is called Wyeth's modification of Gowan's exsector), the jagged end of the upper fragment was removed, the ligaments were severed, and by flexing the knee, the lower fragment rose from its bed; the end of this fragment was then also removed. With a bone drill, an opening was soon made through the ends of the now freshened fragments sufficiently large to admit a silver wire; the bones were approximated, and the wire tied. We then felt that the serious part of our work had been accomplished, and experienced that blessed feeling of serenity that none but the tired and successful surgeon can feel when, the crisis passed, his patient begins the period of recovery. After drying the wound, ligating all bleeding vessels, silk sutures were adjusted, a dressing applied, an opening left through which to dress and inspect the wound; the leg was then placed on an inclined plane, and the patient removed to his ward. He suffered some from pain, and experienced an elevation of temperature. This all subsided, however, and, on the fifteenth day, a permanent silicate of soda dressing was adjusted. At the expiration of the fourth week, the observation was made that union had taken place. The case was unmarked from this date, and when all dressings were removed, a large mass of callous had collected, and union was perfect.

In performing the operation of resection, there are two dangers to be avoided: the first is to be very careful to prevent injuring the periosteum, and if this instrument, Wyeth's Modified Exsector, is used, we must remember to hold the distal end of the fragment with the forceps of the instrument, and saw the bone and instrument off together. I might illustrate by referring to the man who climbed the tree to saw the limbs off, and unthinkingly sawed himself off.

In conclusion, I would like to exhibit an instrument known as a chain saw, used by my father, Dr. D. F. Stuart, in resecting a radius and ulna of a soldier wounded in Georgia, in 1863. After the man was wounded, the instrument was devised and made. This saw was constructed with a file and punch, from a lady's hoop skirt; was designed by Dr. Stuart, and made by a Yankee prisoner.

To-day, for the first time since his recovery from the operation, Dr. Stewart heard from the patient. With tears in his eyes he exclaimed: "God bless my old soldier surgeon; I am glad to know that he is still alive, and I expect to grasp his hand once more at the reunion in May."

